# 499. Immunotherapy with Pattern Recognition Receptor Agonists Improves Morbidity and Mortality in a Corticosteroid-Immunosuppressed Murine Model of Influenza-Associated Pulmonary Aspergillosis

**DOI:** 10.1093/ofid/ofac492.556

**Published:** 2022-12-15

**Authors:** Sebastian Wurster, Jezreel Pantaleón García, Nathaniel D Albert, Scott Evans, Dimiitrios P Kontoyiannis

**Affiliations:** The University of Texas MD Anderson Cancer Center, Houston, Texas; The University of Texas MD Anderson Cancer Center, Houston, Texas; The University of Texas MD Anderson Cancer Center, Houston, Texas; The University of Texas MD Anderson Cancer Center, Houston, Texas; The University of Texas MD Anderson Cancer Center, Houston, Texas

## Abstract

**Background:**

Corticosteroids are known to increase the incidence and worsen the outcomes of influenza-associated pulmonary aspergillosis (IAPA) in patients with severe influenza pneumonia. Adjunct immunomodulatory strategies might improve the treatment of this emerging entity, but i*n-vivo* data in validated corticosteroid-immunosuppressed animal models of IAPA is scarce.

**Methods:**

8-10-week-old female BALB/c mice were infected with 7.5% LD_90_ of a mouse-adapted influenza A/H3N2/Hong Kong/1968 strain, delivered by aerosolization on day (d) 0. On d5 and d8, mice received two intraperitoneal injections of 10 mg cortisone acetate (CA) or mock injections. On d9, mice were intranasally challenged with 50,000 *Aspergillus fumigatus* (AF) conidia. Infection severity was scored using the viral pneumonia score (VPS, 0 = healthy to 12 = moribund) and the murine sepsis score (MSS, 0 = healthy to 3 = moribund). For therapeutic studies, mice received a 30-minute nebulization of the toll-like receptor (TLR) 2/6/9 agonists Pam2+ODN (PMID: 34153197; 4 µM/1 µM) or PBS (mock treatment) on d8 (single-dose) or d8+12 (dual-dose). Therapeutic studies used a combined morbidity/mortality endpoint, with an event defined as either death or reaching a VPS ≥ 7.

**Results:**

CA-immunosuppressed mice with IAPA had 64% 16-day mortality and survivors had severe morbidity (median MSS 2.7). In contrast, all non-immunosuppressed mice with IAPA survived and showed consistently low morbidity (median d16 MSS 0.5). All mock-treated CA-immunosuppressed mice with IAPA reached the combined morbidity/mortality endpoint by d11. Single-dose Pam2+ODN treatment led to universal event-free survival until d13 but all mice deteriorated by d16. Dual-dose Pam2+ODN treatment before and after AF infection led to 75% event-free survival until d21, with all survivors fully recovering (VPS = 0, p < 0.001, **Figure 1**).

**Figure 1:**
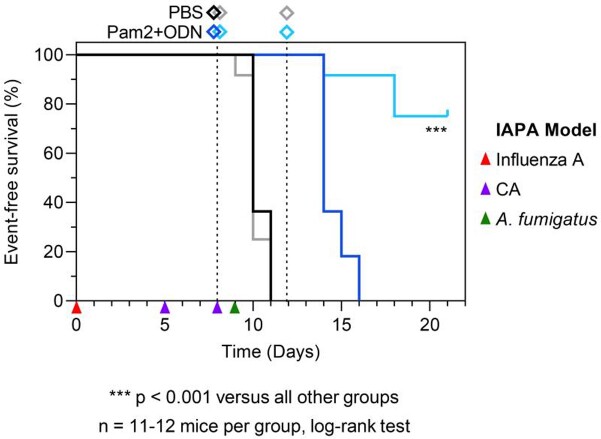

**Conclusion:**

Aligned with clinical evidence, we found detrimental outcomes of IAPA in CA-immunosuppressed mice. Treatment with TLR agonists significantly improved morbidity and mortality of CA-immunosuppressed mice with IAPA. Next, we will study the impact of synergistic anti-infective and immunotherapeutic interventions on the course and immunopathogenesis of IAPA.

**Disclosures:**

**Dimiitrios P. Kontoyiannis, MD, ScD, PhD (hon)**, AbbVie: Advisor/Consultant|Astellas Pharma: Advisor/Consultant|Astellas Pharma: Grant/Research Support|Astellas Pharma: Honoraria|Cidara Therapeutics: Advisor/Consultant|Gilead Sciences: Advisor/Consultant|Gilead Sciences: Grant/Research Support|Gilead Sciences: Honoraria|Merck: Advisor/Consultant.

